# MicroRNA-34a Modulates MDM4 Expression via a Target Site in the Open Reading Frame

**DOI:** 10.1371/journal.pone.0042034

**Published:** 2012-08-01

**Authors:** Pooja Mandke, Nicholas Wyatt, Jillian Fraser, Benjamin Bates, Steven J. Berberich, Michael P. Markey

**Affiliations:** Department of Biochemistry and Molecular Biology, Wright State University, Dayton, Ohio, United States of America; Wayne State University, United States of America

## Abstract

**Background:**

MDM4, also called MDMX or HDMX in humans, is an important negative regulator of the p53 tumor suppressor. MDM4 is overexpressed in about 17% of all cancers and more frequently in some types, such as colon cancer or retinoblastoma. MDM4 is known to be post-translationally regulated by MDM2-mediated ubiquitination to decrease its protein levels in response to genotoxic stress, resulting in accumulation and activation of p53. At the transcriptional level, MDM4 gene regulation has been less clearly understood. We have reported that DNA damage triggers loss of MDM4 mRNA and a concurrent increase in p53 activity. These experiments attempt to determine a mechanism for down-regulation of MDM4 mRNA.

**Methodology/Principal Findings:**

Here we report that MDM4 mRNA is a target of hsa-mir-34a (miR-34a). MDM4 mRNA contains a lengthy 3′ untranslated region; however, we find that it is a miR-34a site within the open reading frame (ORF) of exon 11 that is responsible for the repression. Overexpression of miR-34a, but not a mutant miR-34a, is sufficient to decrease MDM4 mRNA levels to an extent identical to those of known miR-34a target genes. Likewise, MDM4 protein levels are decreased by miR-34a overexpression. Inhibition of endogenous miR-34a increased expression of miR-34a target genes and MDM4. A portion of MDM4 exon 11 containing this 8mer-A1 miR-34a site fused to a luciferase reporter gene is sufficient to confer responsiveness, being inhibited by additional expression of exogenous mir-34a and activated by inhibition of miR-34a.

**Conclusions/Significance:**

These data establish a mechanism for the observed DNA damage-induced negative regulation of MDM4 and potentially provide a novel means to manipulate MDM4 expression without introducing DNA damage.

## Introduction

The gene MDM4 has become a target of interest for therapeutic intervention in cancer. MDM4 serves as an important negative regulator of the p53 tumor suppressor. Through the RING domain at the C-terminus, MDM4 binds p53 and inhibits its ability to transcriptionally regulate gene expression. Recently, MDM4 has been shown to play an additional role in apoptosis by acting as a scaffold at mitochondria to bring together p53 and BCL2 and promote apoptosis [1]. The importance of MDM4 in human cancer is underscored by its frequent amplification in certain tumor types, such as colon cancer [2], gliomas [3]–[5] and retinoblastomas [6]. Full activation of p53 in response to DNA damage requires inhibition of MDM4 [7]. Targeting of MDM4 represents an attractive therapeutic approach for the reactivation of p53, especially given that restoration of p53 in the absence of MDM4 is not lethal to normal cells [8]. It is therefore important that we understand the mechanisms controlling MDM4 activity.

MDM4 has long been understood as a target of the closely-related protein MDM2. MDM2 acts an E3 ubiquitin ligase, targeting MDM4 protein for degradation during the DNA damage response [9], [10]. Localization of MDM4 to the nucleus is also regulated, in part by p53 and MDM2, but potentially by other proteins as well [11], [12]. Recently, MDM4 was shown to bind to the noncoding 5S rRNA [13]. This stabilizes MDM4 by inhibiting the ability of MDM2 to ubiquitinate MDM4.

Transcriptionally, MDM4 is controlled by MAPK signaling through the transcription factors c-Ets and Elk-1 [14]. Several truncated alternative transcripts of MDM4 have been identified, some of which have been shown to influence p53 activity in cancer cells (reviewed in [15]). A recent report has shown a longer alternative transcript of MDM4, termed HDMX-L, which interestingly is induced by p53 from a p53 binding site between exon 1 of the MDM4 gene and the alternative exon 1β [16]. However, full-length MDM4 mRNA transcripts have been found to decrease in response to damage, independent of p53 status [17]. These seemingly contradictory reports have been thus far explained by differences in the doses of DNA-damaging agents used between the two studies. Importantly, a mechanism for decreased MDM4 mRNA has not been demonstrated. This was the aim of the experiments detailed here.

MicroRNAs are short noncoding RNAs that interfere with gene expression by binding to imperfectly complimentary mRNAs, inducing their destruction and/or inhibiting their translation. miR-34a has been demonstrated to be robustly induced directly by p53 [18]–[22] and contribute to the pro-apoptotic effect of p53 by down-regulating genes involved in cell survival and proliferation (reviewed in [23], [24]). Induction of miR-34a has been previously shown to correspond to the decrease in MDM4 mRNA following DNA damage in several cell lines [17]. Targeting of MDM4 by miR-34a would be consistent with the pro-apoptotic effect of miR-34a expression.

Here, we demonstrate that MDM4 mRNA is targeted by the microRNA miR-34a. Expression of miR-34a varies greatly between cell lines. Over expression of miR-34a in cells with low endogenous levels can inhibit the expression of endogenous MDM4 mRNA and protein. Importantly, these effects do not seem to be mediated by the 3′ untranslated region (UTR) of MDM4. Rather, a miR-34a site in the coding region of the last exon of MDM4 (exon 11) is sufficient to influence reporter gene expression.

## Results

### miR-34a and MDM4 are Differentially Expressed in Human Cell Lines

**Figure 1:**
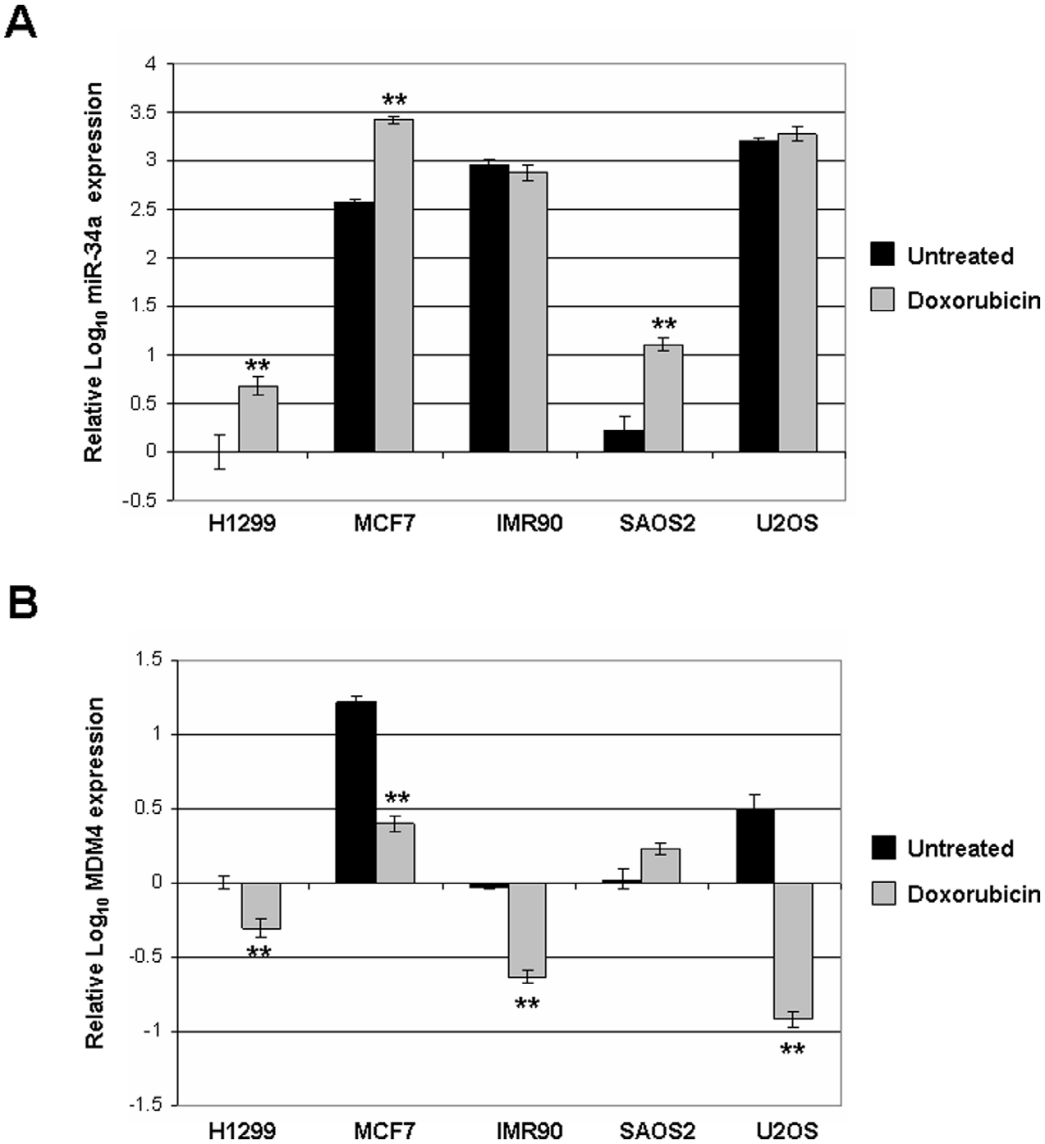
MDM4 and miR-34a are differently expressed in human cell lines. (A) Real-time quantitative PCR was performed in quadruplicate for miR-34a using RNA extracted from the indicated cell lines before and after treatment with 0.5 ug/ml doxorubicin for 24 hours. Y-axis is log base 10. Error bars show 95% confidence intervals. Double asterisks indicate paired, one-tailed t-test values <0.01 comparing the untreated to doxorubicin treated condition for each cell line. (B) RT-qPCR as before, for MDM4.

The tumor suppressor p53 is lost or mutated in about half of human cancers [25]. In most if not all of the rest, p53 is inactivated or held at low levels by other means. In some cancers this is achieved through overexpression of MDM4, which binds p53 and prevents transcription of p53 target genes. The frequency of MDM4 overexpression varies by cancer type; for example, a majority of retinoblastomas show amplification of MDM4 [6], but it is very uncommon in prostate cancer [26]. In order to explore the correlation between MDM4 expression and miR-34a expression, we surveyed a variety of cell lines for expression of each (Figure 1). The non-small cell lung carcinoma line H1299, in which p53 is homozygously deleted, had the lowest expression of both MDM4 and miR-34a, although MDM4 levels could still be reduced by treatment with doxorubicin. SAOS2 osteosarcoma cells, also p53-null, had similarly low endogenous miR-34a and MDM4. SAOS2 was the only line tested in which MDM4 was slightly induced following DNA damage. Interestingly, miR-34a remains inducible in SAOS2, indicating alternative controls on miR-34a expression. In p53-positive cell lines MCF7 (breast cancer) and U2OS (another osteosarcoma), miR-34a was highly expressed; however, it was only able to be further induced (approximately 10-fold) upon DNA damage in MCF7. Both show a robust down-regulation of MDM4 following damage. Human primary fibroblasts IMR90 showed high expression of miR-34a similar to MCF7 and U2OS, no induction of miR-34a following DNA damage, and relatively low levels of MDM4 which still decrease after damage. This is likely due to an alternative splicing of the MDM4 primary transcript demonstrated previously in these cells [17]. Taken together, these data indicate that different mechanisms may play greater or lesser roles in controlling MDM4 expression in different cell lines.

### Over Expression of miR-34a Inhibits Expression of known Targets and MDM4

**Figure 2:**
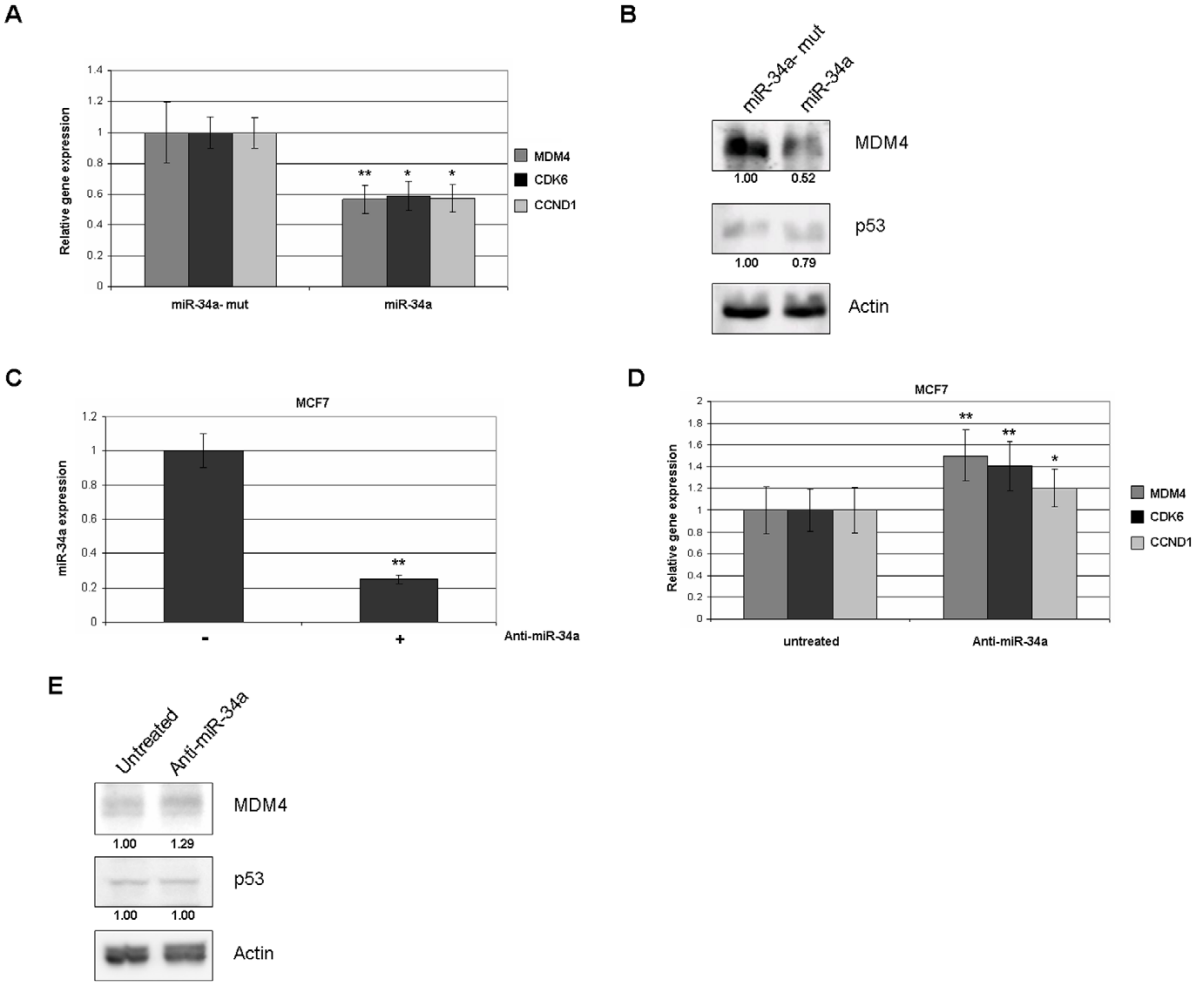
Endogenous MDM4 is repressed by miR-34a. (A) MCF7 cells were transfected with expression plasmids for miR-34a or a control mutant of miR-34a. Total RNA was extracted after 48 hours, and RT-qPCR was used to quantify the expression of MDM4 and the known miR-34a target genes CDK6 and CCND1. Expression is relative to the control (miR-34a-mut) transfection condition. Data are the averages of at least three independent experiments. Error bars show 95% confidence intervals. Asterisks and double asterisks indicate t-test values <0.05 and <0.01, respectively, comparing the miR-34a expression plasmid to the control mutant. (B) MCF7 cells were transfected as in (A), and whole cell lysates used for immunoblots to detect MDM4, p53, and beta-actin. Quantification relative to the 34a-mutant condition and normalized to actin are shown below each panel. (C and D) MCF7 cells were transfected with an inhibitor of miR-34a (anti-miR-34a) for 48 hours. RT-qPCR was performed for miR-34a, MDM4, and known miR-34a target genes. (E) Following transfection with miR-34a inhibitor as before, total protein was extracted and immunoblots were performed for the indicated proteins in MCF7.

In order to explore the effect of miR-34a on MDM4 expression, we used a cell line with high endogenous MDM4 and miR-34a (MCF7). In MCF7 cells miR34a could be induced and MDM4 could be repressed following DNA damage by doxorubicin treatment (Figure 1). These cells were transiently transfected with an expression plasmid encoding either miR-34a or a mutated miR-34a that does not produce a mature microRNA [21]. Overexpression of miR-34a in MCF7 (Figure 2A) resulted in 40% repression of MDM4 observed by RT-qPCR. Importantly, this decrease matched closely in magnitude the repression of the known miR-34a target genes CCND1 and CDK6 [27]. Similar results were observed in H1299, a cell line with low endogenous miR-34a (not shown), suggesting the effect of miR-34a was p53 independent. Overexpression of miR-34a similarly leds to a decrease in MDM4 protein expression (Figure 2B).

In order to determine whether endogenous miR-34a inhibits MDM4, MCF7 cells were transfected with an inhibitor of miR-34a (mirVana miRNA inhibitor, Invitrogen). A reduction of nearly 80% in miR-34a levels was achieved (Figure 2C), although this may still represent relatively high levels of miR-34a compared to other cell lines (Figure 1A). Interestingly, inhibition of miR-34a in MCF7 cells was able to de-repress MDM4, CCND1, or CDK6 (Figure 2D). A similar slight increase in MDM4 protein expression was observed (Figure 2E).

### The 3′ Untranslated Region of MDM4 does not Respond to miR-34a

**Figure 3:**
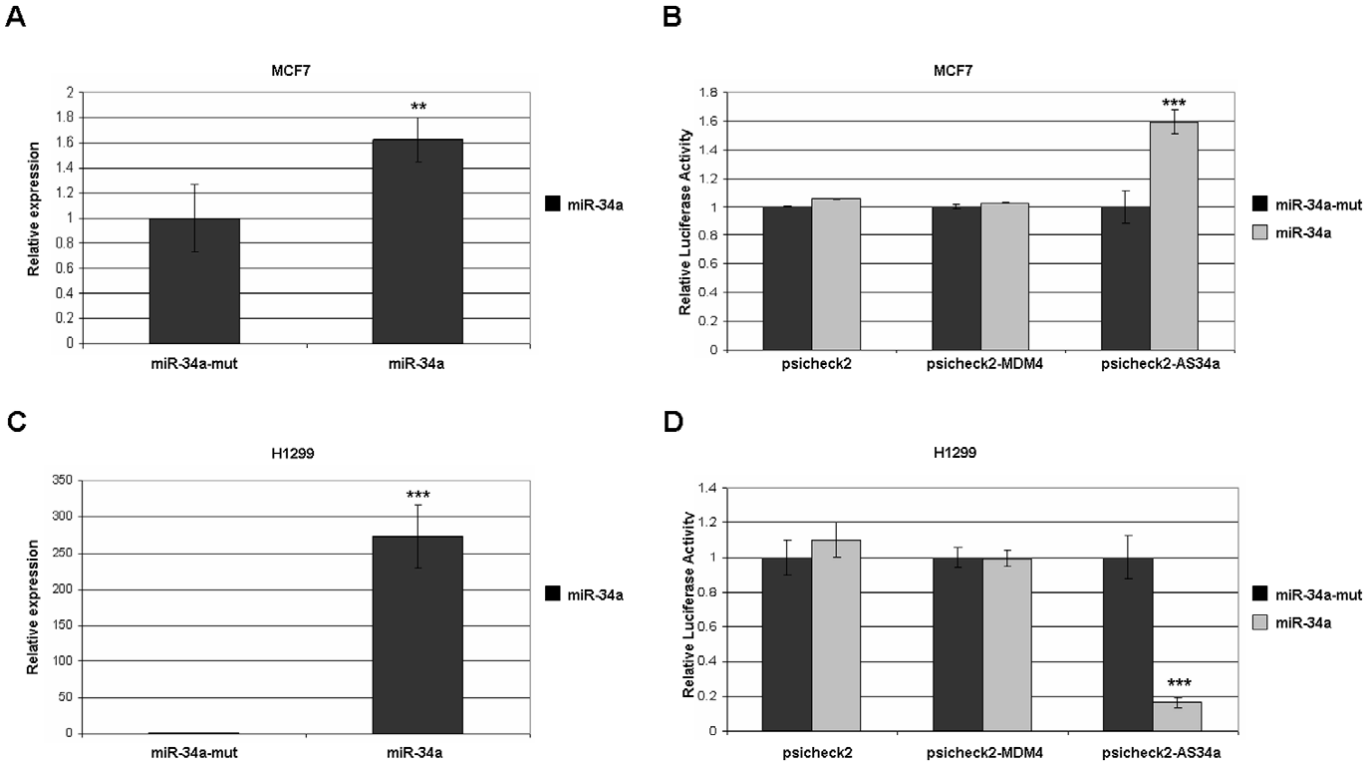
The 3′ UTR of MDM4 is unresponsive to miR-34a expression. Data are the averages of at least four independent experiments, with standard deviation indicated in error bars. Double asterisks indicate paired, one-tailed t-test values <0.01 between control and experimental conditions. Triple asterisks indicate p-value <0.001. (A) MCF7 cells were co-transfected with an expression plasmid for miR-34a or the nonfunctional mutant miR-34a-mut which produces no mature miR-34a transcript [21]. Expression of miR-34a was determined by RT-qPCR. (B) Expression plasmids for miR-34a or miR-34a-mut were co-transfected with a luciferase reporter plasmid containing approximately 1700 bp of the MDM4 3′UTR downstream of luciferase. Cell lysates were used for luciferase assays 48 hours after transfection. Expression is relative to the expression of luciferase in the psicheck2 vector lacking a 3′ UTR. The plasmid psicheck2-AS34a is a positive control for regulation by miR-34a and contains a miR-34a response element [51]. (C and D) As above, for H1299 cells.

MicroRNAs are known to bind at the 3′ UTR to down-regulate target mRNAs, through degradation of the mRNA and/or inhibition of translation. To test for regulation of MDM4 by miR-34a, we initially created a luciferase reporter construct based on the NCBI reference entry NM_002393.2 containing approximately 800 bp of the MDM4 3′ UTR. This region showed no response to miR-34a overexpression in MCF7, H1299, or HCT116 (data not shown). When the NCBI reference entry for MDM4 was updated to NM_002393.3, the 3′UTR was indicated to be much larger that the 800 bp being examined. In order to include all the potential miR-34a sites predicted earlier [17], a second construct containing approximately 1700 bp of the 3′ UTR was generated in order to capture the region responsible for binding miR-34a. This reporter was cotransfected into MCF7 with expression plasmids for miR-34a or miR-34a-mut. Transfection resulted in approximately 60% increase in miR-34a expression compared to endogenous miR-34a levels (Figure 3A), but no change in reporter activity (Figure 3B). To achieve a greater overexpression of miR-34a, the experiment was repeated in H1299. Low endogenous miR-34a allowed transfection to achieve 250-fold increase in miR-34a expression (Figure 3C). However, this region remained completely unresponsive to miR-34a expression in H1299 (Figure 3D).

### The MDM4 Open Reading Frame Contains a Functional miR-34a Site

**Figure 4:**
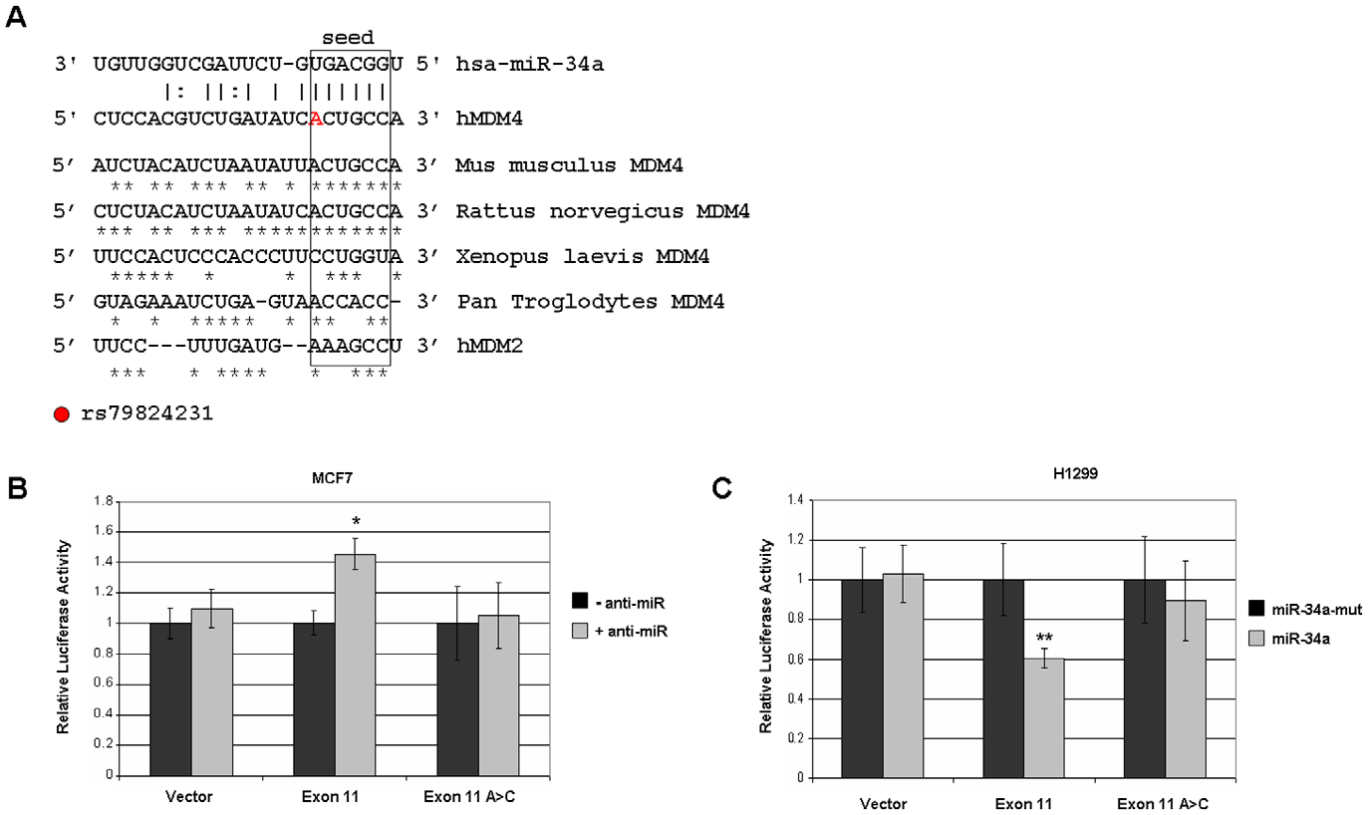
The MDM4 open reading frame contains a functional miR-34a responsive site. (A) Binding site for miR-34a predicted in human MDM4 mRNA by miRanda. Binding is indicated by solid lines, while wobble base pairing is indicated by a dashed line. Identity with the human MDM4 sequence is indicated by asterisks. The seed region of miR-34a, positions 2–7, are boxed. The homologous region of human MDM2 mRNA is also shown for comparison. (B) In MCF7 cells, the miR-34a inhibitor was cotransfected with a reporter gene containing the ORF miR-34a site from MDM4 (“Exon 11″) or the reporter with the rs79824231 SNP (“Exon 11 A>C”). Data are the averages of at least six independent experiments, with standard deviation indicated in error bars. Asterisks indicate t-test values <0.5. (C) In H1299 cells, the reporters were cotransfected with an expression plasmid for miR-34a or miR-34a-mut. Data are the averages of at least six independent experiments, with standard deviation indicated in error bars. Double asterisks indicates paired, one-tailed t-test value <0.01.

To determine whether the observed repression of endogenous MDM4 was an indirect effect, the 3′ UTR of MDM4 was reexamined with improved microRNA site prediction software. The August 2010 release of miRanda [28] did not identify any of the miR-34a sites suspected previously in the 3′ UTR of MDM4. However, a very strong candidate site was predicted just upstream of the 3′ UTR, within the protein coding region of exon 11 (Figure 4A). This site has an exact match to the miR-34a seed site at position 2–8 and an A in position 1, defining an 8mer-A1 site [29]. This also matches the seed sequence used by CDK6 and CCND1. Although exon 11 encodes the C-terminal RING domain of MDM4, and this region is also found in the related protein MDM2, it is interesting to note that MDM2 contains a different codon at the homologous region which destroys the miR-34a seed sequence. This locus, especially the miR-34a seed region, is fairly well conserved in MDM4 from other species, with the chimpanzee and *Xenopus* genes being the only exceptions in the sequences we compared (Figure 4A). The seed sequence in human MDM4 is also potentially disrupted by a single nucleotide polymorphism (SNP, rs79824231) of currently unknown frequency in the human population.

To determine whether this ORF site is functional, the predicted miR-34a site was inserted downstream of luciferase in a reporter vector. In MCF7 cells with high endogenous miR-34a, knockdown of miR-34a (Figure 2D) resulted in an increase in reporter activity (Figure 4B). Conversely, when transfected into H1299 cells with low endogenous miR-34a, luciferase expression was significantly repressed by miR-34a overexpression (Figure 4C). Thus, the miR-34a site in the coding region of MDM4 exon 11 is functional and responsive to miR-34a. Interestingly, all responsiveness to miR-34a can be abolished by the A to C transversion found in rs79824231. The A allele was confirmed by sequencing in all cell lines used in this study.

## Discussion

Many factors influence the expression of MDM4. Copy number can be increased or copies lost, and transcription can be influenced by known transcription factors. Alternative splicing influences the types of MDM4 transcripts produced, and p53 can induce at least one alternative form in particular. Many post-translational effects influence MDM4 protein stability and localization. Importantly, ubiquitination by MDM2 and deubiquitination by HAUSP or USP2a play a major role in MDM4 stability [30], [31]. Even the noncoding 5S rRNA has been shown to bind MDM4 and inhibit ubiquitination [13]. To date, two microRNAs have been shown to influence MDM4 expression. First, mRNA from the C allele but not the majority A allele of rs4245739 (a SNP in the 3′ UTR of MDM4) has been demonstrated to be decreased by miR-191, and hence the A allele is associated with high-grade carcinomas and higher expression of MDM4 [32]. Second, high expression of miR-10a in acute myeloid leukemia has been shown to associate strongly with negative regulation of MDM4, and to repress an MDM4 3′ UTR reporter [33]. It may be that these or yet other microRNAs act to repress MDM4 in response to DNA damage in some cell lines.

Previous results have shown a decrease in expression of full-length MDM4 mRNA (flMDM4) in immortal and primary cell lines following treatment with DNA damaging agents doxorubicin or cisplatin [17]. It was speculated that multiple factors contributed to this decrease. First, DNA damage was observed to increase the expression of an alternative transcript of MDM4, XAlt2. Importantly, this increase was not stoichometrically identical to the decrease in flMDM4. Second, flMDM4 mRNA was seen to be less stable under DNA damage conditions, which implicated a possible role for a miRNA targeting flMDM4. Last, in MCF7 cells the decrease in flMDM4 was observed to correlate with an increase in miR-34a expression. These data are consistent with recent data demonstrating a decrease in MDM4 mRNA following targeting of multiple components of the splicing machinery [34]. Interestingly, this decrease in MDM4 mRNA did not depend on alternative splicing of MDM4 primary transcripts, underlining the role of additional mechanisms controlling MDM4 mRNA levels (ibid). A mechanism for regulation of MDM4 by miR-34a, however, remained to be determined. At the same time, it has been reported that the MDM4 gene is transcriptionally activated by p53 itself, through a p53-responsive promoter in the first exon of the MDM4 gene [16], which is in effect similar to the targeting of MDM2 by p53 [35]. The reason for these contradictions remains to be defined; however, in our hands we have yet to observe a change in the low levels of HDMX-L transcripts we observe under the conditions used in its discovery (cell line, drug dosages, and PCR primers).

The current data support a role for miR-34a in the regulation of MDM4 expression through targeting a site within the MDM4 open reading frame. Potential miR-34a binding sites in the 3′ UTR of MDM4 were examined and found not to represent functional loci. Indeed, more recent and improved *in silico* prediction methods for miRNA sites failed to identify miR-34a sites in the MDM4 3′ UTR that were previously suspected [17]. Rather, a strong prediction was made within the ORF of the final exon of MDM4. miRNA recognition of a target mRNA through the coding region is not without precedent. Several splice variants of the human gene DNMT1 have been shown to be regulated by miR-148 via a site in the protein coding region [36], and this type of targeting is common in plants [37], [38]. Another example from a human gene is human hepatocyte nuclear factor 4 alpha (HNF4A), which is regulated by a miR-34a site in the ORF of that gene [39]. Moreover, many functional ORF miRNA sites have been detected, and mRNAs with an 8mer site in the ORF were shown to be repressed by a microRNA significantly better than mRNAs without a site [40]. Fang and Rajewsky recently observed that mRNA repression is achieved *in vivo* even with only microRNA seed sites in the coding region, although these synergize with 3′ UTR seed sites [41]. This MDM4 ORF site is of the most highly conserved type, 8mer-Al, as defined by Jan et al. [29]. Accordingly, the miRSVR score -1.286 corresponds to approximately the top 2.5% of all predicted miRNA sites [28]. The predicted seed region exactly matches that used by the known miR-34a targets CDK6 and CCND1 [27].

This miR-34a site is expected to be present in all transcript variants of human MDM4 described to date. Exon 11 is present in MDM4-211 [42], MDM4-A [43], MDM4-G [43], and MDM4-ALT2 [44]. Even in the known transcripts with premature termination codons, MDM4-S [45], [46] and MDM4-ALT1 [44], the mRNA retains exon 11.

Interestingly, this miR-34a site in the ORF of MDM4 exon 11 is also the site of a human single nucleotide polymorphism (SNP). This site, rs79824231, is an A>C missense mutation which changes the codon from threonine to proline. It was identified in the 1000 Genomes Project [47], and frequency data in human populations remains to be determined. A second SNP two bases upstream, rs80287024 (T>C), was recently withdrawn by the 1000 Genomes Project due to a high false positive rate. Nevertheless, we genotyped both of these sites in the cell lines MCF7, H1299, SAOS2, and HCT116. The majority A allele was found each case. From the location of rs79824231 within the seed region used by miR-34a, the C allele would be expected to disrupt recognition by miR-34a, and indeed this was our finding by reporter assays (Figure 4C and D). The C allele would then be expected to be present in a subset of cell lines in which MDM4 does not respond to miR-34a. A similar phenomenon has been described in HNF4A, which also has a miR-34a site in the ORF destroyed by a SNP [48].

It has been previously demonstrated that p53 induces the expression of miR-34a [18]–[22], and that miR-34a contributes to the pro-apototic effect of p53. In turn, miR-34a activates p53 by inhibiting the deacetylase SIRT1 [49]. Without SIRT1 deacetylation of p53 at K382, p53 pro-apoptotic activity remains uncompromised [50]. Data presented here are consistent with a pro-apoptotic effect of miR-34a on p53, as miR-34a would inhibit expression of a negative regulator of p53.

## Materials and Methods

### Cell Culture

Colon carcinoma cell line HCT116 and human non-small cell lung carcinoma cell line H1299 were cultured in Dulbecco’s modified Eagle’s medium (DMEM, Atlanta Biologicals) supplemented with 10% normal calf serum. Breast cancer cell line MCF7, IMR90 human fibroblasts, and SAOS2 and U2OS osteosarcoma cell lines were cultured in DMEM supplemented with 10% fetal calf serum. All cell lines were obtained from the American Type Culture Collection.

### Quantitative RT-PCR

H1299, MCF7, IMR90, SAOS2, and U2OS were treated with doxorubicin at 0.5 µg/ml for 24 hours prior to RNA extraction using EZNA Total RNA Extraction kit and protocols (Omega Bio-Tek). 500 ng of total RNA was reverse transcribed, then diluted to 2.5 ng per qPCR reaction using the TaqMan Assay on Demand system, as previously described [17].

MCF7 cells were transfected 48 hours after seeding (2×10^5^) cells in 6 cm plates. 30 nM miRVANA hsa-miR-34a inhibitor (Ambion) was added to the serum free media. 6 µl lipofectamine2000 was added to 50 µl serum free media. The two solutions were mixed in equal quantities and allowed to stand at room temperature for 20 minutes. 100 µl of the resulting solution was added to 6 cm plates containing 2.9 ml serum free media. After 5 hours of incubation the cultures were returned to DMEM media containing 10% serum. 48 hours later the cultures were processed for RNA extraction.

For RT-qPCR of microRNA, RNA extraction was carried out using the TRIZOL method according to the manufacturer’s protocol. The RNA obtained by this method was further processed to enrich for microRNA using the RT^2^qPCR Grade miRNA Isolation Kit (SAbiosciences) according to manufacturer’s protocol. 10 ng of the microRNA was reverse transcribed using miRNA-specific primers for hsa-miR-34a or hsa-mir213 (to normalize) according to the TaqMan® MicroRNA Reverse Transcription Kit (Applied Biosystems). qPCR was performed using TaqMan® Universal PCR Master Mix (Applied Biosystems) and the suggested protocols.

### Reporter Assays

H1299 cells were transfected 24 hours after seeding (2.5×10^6^) cells in 6 cm plates. The psicheck2 vector or psicheck2-ex11 reporter or psichcheck2-ex11 A>C reporter, and mir-34a expression plasmid or mir-34a-mut expression plasmid, were added to serum free media (final volume of 200 µl). 6µl of lipofectamine2000 was added to 200 µl of serum free media, which was then mixed with the plasmid DNA and incubated 30 minutes at room temperature. 400 µl of the resulting DNA:lipid solutions were added to cells on 6 cm plates containing 2.5 ml serum free media. After 5 hours of incubation the cultures were replaced with DMEM media containing 10% NCS. 48 hours later the cultures were processed for the dual luciferase assay using the Promega Dual Luciferase assay system and protocol. Samples were normalized to luciferase expression in the vector-transfected cells. Paired, one-tailed t-tests were used to determine significance. The plasmid psicheck2-AS34a contains a consensus response site for miR-34a, and was used as a positive control for response to miR-34a overexpression or inhibition. It has been described [51].

MCF7 cells were transfected 24 hours after seeding the 24-well plates (4×10^4^ cells/well). The reporter plasmid (0.2 µg) with or without the hsa-miR-34a inhibitor (30 nM) and the psicheck2 (vector) plasmid (0.2 µg) with or without the inhibitor (30 nM) were added to 50 µl of serum free DMEM media. 2 µl of lipofectamine2000 was added to 50 µl serum free DMEM media. The two solutions were mixed in equal quantities and allowed to stand at room temperature for 20 minutes. 100 µl of the resulting DNA:lipid solution was added to culture plates containing 2.9 ml serum free media. After 5 hours of incubation the serum free media was replaced with DMEM media containing 10% FBS. 48 hours later the cells were processed for the Dual Luciferase assay (Promega).

### Immunoblotting

MCF7 cells were transfected 24 hours after seeding the 6 cm plates (2×10^4^ cells) and treated with 30 nM miRVANA hsa-miR-34a inhibitor (Ambion) as described above. 48 hours later the cells were processed for protein extraction.

The cells were harvested from the 6 cm plates in phosphate buffer saline (PBS) and lysed in RIPA buffer (50 mM Tris pH = 8.0, 150 mM NaCl, 1% NP-40, 0.5% Deoxycholate, 0.1% SDS) (150 ul) to which was added protease inhibitor cocktail PIC (1 µl/100 µl) and NaF (to 50 µM). The contents were mixed by pipeting, incubated on ice for 30 minutes and centrifuged at 10,000 rpm for 5 minutes at 4°C. The supernatant was then taken and protein concentration measured using the BioRad Protein Assay. The proteins were separated by SDS-PAGE. After separation the proteins were transferred onto a PVDF membrane (at 400 milliamps for 1 hour). This was followed by blocking with 5% milk in Tris-buffered saline-tween 20 (TBST) for one hour and probing with the relevant primary antibody overnight. Following 2 thirty minute washes with TBST, a horse radish peroxidase (HRP) conjugated secondary antibody was added for 1 hour and the protein band was observed following addition of Super signal West Pico chemiluminescent substrate (Thermo Scientific). The following antibodies were used: HdmX/Mdm4 A300-287A (Bethyl labs), actin AC-40 (Sigma), p53 FL393 (Santa Cruz Technology), anti-rabbit IgG HRP (Promega), anti-mouse IgG HRP (Promega).

Relative quantification of western blots was performed with ImageJ [52]. Values shown are relative to the control condition and normalized to actin, to control for any protein loading inequalities.

### Cloning

miR-34a sites were predicted by the August 2010 release of miRanda [53]. For the plasmid “psicheck2-MDM4”, the 3′ UTR region following the stop codon of MDM4 to 1,735 bp downstream was amplified by PCR from BAC 433N15 (BACPAC Resources Center) and inserted between SgfI and NotI sites in the psicheck2 reporter plasmid (Promega) downstream of *renilla* luciferase. Cloning primers were: sense 5′- AAA AAA GCG ATC GCA ATG CAT TTA TTC CGT TCA CTT -3′ and antisense 5′- AAA AAA GCG GCC GCG GTG TAA GCA GCT CCA GAG G -3′. For the plasmid “psicheck2 Exon 11”, a 444 bp region of the coding region of exon 11 of the human MDM4 gene, surrounding the putative ORF miR-34a site, was amplified by PCR from BAC 433N15 (BACPAC Resources Center) and inserted between SgfI and NotI sites in the psicheck2 reporter plasmid (Promega) downstream of *renilla* luciferase. Cloning primers were: sense 5′- AAA AAA GCG CCT TGA GGA AGG ATT GGT AT -3′ and antisense 5′- AAA AAA GCG GCC GCA GCC CCA GCC TTC TTT AGT C -3′. Identity of all clones was verified by sequencing.

The plasmid “psicheck2 exon 11 A>C” was generated by site-directed mutagenesis of the “psicheck2 exon 11” plasmid according to the QuikChange II Site-Directed Mutagenesis kit and protocol (Aligent Technologies). An A to C mutation was generated to correspond to the human SNP rs79824231, which lies in the seed region of the exon 11 ORF miR-34a site. Mutagenic primers were: sense 5′- CTC TCC ACG TCT GAT ATC CCT GCC AT ACCT GAA AA -3′ and antisense 5′- TTT TCA GGT ATG GCA GGG ATA TCA GAC GTG GAG AG -3′. Mutation of the target site was verified by sequencing.

### Sequence Alignment

Sequences were retrieved from GenBank and aligned using ClustalW2 v2.1 [54] with default (slow) alignment settings. The accession and version numbers for sequences used were: *Mus musculus* EU568360.1, *Rattus norvegicus* NM_001012026.1, *Xenopus laevis* NM_001088965.1, *Pan troglodytes* XM_003308706.1, human MDM4 NM_002393, and human MDM2 NM_002392.3.
